# Implications for Diverse Functions of the LINC Complexes Based on the Structure

**DOI:** 10.3390/cells6010003

**Published:** 2017-01-26

**Authors:** Miki Hieda

**Affiliations:** Department of Medical Technology, Ehime Prefectural University of Health Sciences, Ehime 791-2101, Japan; mhieda@eup.ac.jp; Tel.: +81-89-958-2111; Fax: +81-89-958-2177

**Keywords:** LINC complex, SUN proteins, KASH, nesprin proteins, lamin

## Abstract

The linker of nucleoskeleton and cytoskeleton (LINC) complex is composed of the outer and inner nuclear membrane protein families Klarsicht, Anc-1, and Syne homology (KASH), and Sad1 and UNC-84 (SUN) homology domain proteins. Increasing evidence has pointed to diverse functions of the LINC complex, such as in nuclear migration, nuclear integrity, chromosome movement and pairing during meiosis, and mechanotransduction to the genome. In metazoan cells, the nuclear envelope possesses the nuclear lamina, which is a thin meshwork of intermediate filaments known as A-type and B-type lamins and lamin binding proteins. Both of lamins physically interact with the inner nuclear membrane spanning SUN proteins. The nuclear lamina has also been implicated in various functions, including maintenance of nuclear integrity, mechanotransduction, cellular signalling, and heterochromatin dynamics. Thus, it is clear that the LINC complex and nuclear lamins perform diverse but related functions. However, it is unknown whether the LINC complex–lamins interactions are involved in these diverse functions, and their regulation mechanism has thus far been elusive. Recent structural analysis suggested a dynamic nature of the LINC complex component, thus providing an explanation for LINC complex organization. This review, elaborating on the integration of crystallographic and biochemical data, helps to integrate this research to gain a better understanding of the diverse functions of the LINC complex.

## 1. Introduction

The nuclear envelope (NE) is composed of the inner nuclear membrane (INM) and outer nuclear membrane (ONM), which are separated by a 40–50 nm perinuclear space (PNS) and spanned by the nuclear pore complex. More than 100 putative integral membrane proteins have been found to reside in either the INM or ONM [1,2,3,4,5]. The Sad1 and UNC-84 (SUN) homology domain proteins, which are type II INM spanning transmembrane proteins, are widely conserved in all eukaryotes and share a common carboxyl-terminal motif of ~175 amino acids, termed the SUN domain [6], so named for the homology between Sad1 from *Schizosaccharomyces pombe* and UNC-84 from *Caenorhabditis elegans* [7,8,9]. Nematodes and flies possess two genes encoding SUN proteins and yeasts contain only one such gene; however, mammalian SUN proteins are encoded by at least five genes, *SUN1–5*. *SUN1* and *SUN2* are widely expressed in mammalian somatic cells [10,11], whereas *SUN3*, *SUN4* (also known as sperm–associated antigen 4, *SPAG4*), and *SUN5* (also known as SPAG4-like, *SPAGL*) are largely, but not entirely, restricted to the germ cells [12,13,14,15]. SUN proteins are composed of three domains: a transmembrane domain, an amino-terminal nucleoplasmic domain that interacts with lamina and chromatin-binding proteins, and a carboxyl-terminal region that protrudes into the PNS and contains coiled-coil domains and a conserved SUN domain [10,11,16]. Klarsicht, Anc1, and Syne1 homology (KASH) domain proteins are another class of type II integral membrane proteins; however, in contrast to SUN proteins, most of KASH domain proteins reside at the ONM. The carboxyl termini of KASH proteins contain the KASH domain, which is a conserved stretch of ~30 amino acids that typically ends with the highly conserved motif, PPPX, frequently PPPT. The KASH domain extends into the PNS and interacts with the SUN domain of SUN proteins. In contrast, the amino-terminal regions of KASH proteins are exposed in the cytoplasm, where they associate with the cytoskeleton, including actin filaments, microtubule motors, and intermediate filaments [17,18,19,20]. The human genome contains six genes encoding KASH proteins, four of which are nesprins (nesprin-1–4). Nesprin-1, -2, and -3 have multiple isoforms resulting from alternative splicing and transcription initiation sites [21], and are expressed in a wide variety of tissues, whereas nesprin-4 expression is largely restricted to the secretory epithelia [22]. There are some nesprin isoforms that have the KASH domain and are present in the nucleus, probably inserted in the INM [23]. One of the other KASH proteins is KASH5, which is a meiosis-specific KASH protein [24]. Another recently characterized putative KASH protein is lymphoid-restricted membrane protein (lrmp), which is required for nucleus–centrosome attachment and pronuclear congression during fertilization [25]. SUNs and nesprins form a complex via their SUN and KASH domains, respectively. This protein complex physically links the nucleoskeleton and cytoskeleton, and is thus named the linker of nucleoskeleton and cytoskeleton (LINC) complex [11,20]. In metazoan cells, the NE harbours the nuclear lamina, which is a meshwork of intermediate filaments, mainly A-type and B-type lamins and lamin binding proteins. Note that, there is a small but important fraction of nucleoplasmic lamin A. The first evidences for SUN protein binding to lamins came from *C. elegans* [26,27]. Mammalian SUN proteins interact with A-type lamin, whereas their binding to B-type lamins is generally considered to be very weak [11,16]. However, we recently demonstrated that B-type lamins also interact with SUN1 but not with SUN2 [28].

The LINC complex performs diverse functions, including nuclear shaping and positioning [29], maintenance of the centrosome–nucleus connection [30], DNA repair [31,32], nuclear membrane spacing [11], cell migration [28,29,33,34,35], and moving chromosomes within the nucleus during meiosis [36]. In addition, lamins play various roles such as maintenance of nuclear integrity, cell cycle regulation, mechanotransduction, cellular signalling, and DNA repair. Because all of these functions are critical for cell viability, variations in the expression or dysfunction of lamins and their interacting LINC complexes are associated with a wide range of diseases, including muscular dystrophy, cardiomyopathies, lipodystrophy, progeria, cancer, and neurological diseases [37]. Indeed, *LMNA*, encoding A-type laminins, is currently the gene with the greatest number and most diverse forms of disease-linked mutations in the human genome [38].

The LINC complex and nuclear lamins combine to form a solid scaffold from which they carry out their diverse functions. However, it is unknown how cells regulate these multiple functions, and whether the LINC complex–lamins interactions are essential for these diverse functions. There are several reasons contributing to the lack of clarity on these questions. One reason is that mammalian somatic cells express at least two SUN domain proteins, which have partially overlapping functions, and up to four KASH domain partners (i.e., nesprins 1–4). Based on the SUN2/KASH peptide crystal structure, it has been believed that each LINC complex is composed of three SUN and three nesprin molecules. Therefore, a diverse range of LINC complexes is possible, which may relate to their multiple functions. Here, I first review the structure of SUN proteins [39,40,41,42,43] recently revealed by crystallographic analyses, which is consistent with the established biochemical data. I then discuss the possible functions of the dynamic interactions between SUN and lamins. Integrating this recent insight with well-established knowledge of these interactions should help to provide a better foundation for elucidating the regulatory mechanisms of the LINC complex, and help to understand its cellular functions and roles in diseases.

## 2. Overall Structure of SUN Domains

**Figure 1:**
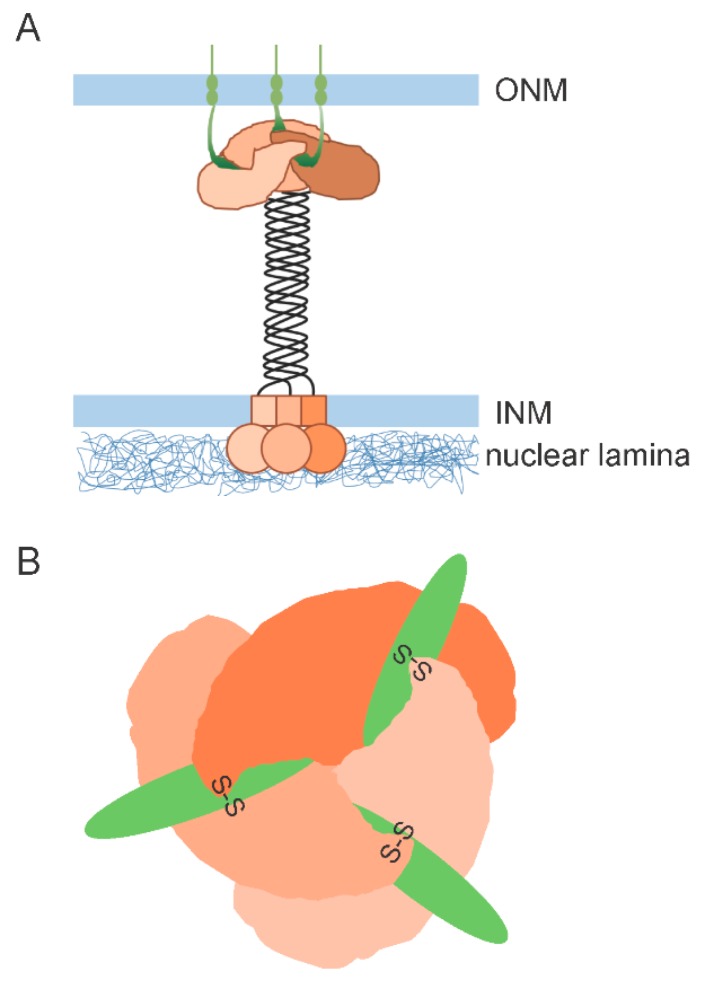
Schematic representation of SUN–KASH interactions based on the results of Sosa et al., and Wang et al. [40,41]. (**A**) A schematic representation of SUN molecular structure with KASH peptide. Orange colour represents SUN molecules and green colour represents KASH and transmembrane domains of nesprin proteins; (**B**) The top-down view of the SUN2/KASH domain. Orange colour shows SUN domain of SUN2 molecules and green colour represents KASH domain of nesprin proteins. Each KASH peptide interacts with the KASH lid of one SUN2 protomer and the beta-sandwich of the neighbouring SUN2 protomer. SUN, Sad1 and UNC-84 homology domain proteins; KASH, Klarsicht, Anc1, and Syne1 homology domain proteins.

The crystallographic structure of the human SUN2_519–716_ protein showed that the SUN domain (SUN2_555–716_) and upstream extension is sufficient to form a homotrimer, which exhibits a perfect three-fold symmetry and resembles a cloverleaf (~65 Å) sitting on a short fragment of a stem (~30 Å of length) [39]. Two subsequent studies revealed the structure of the SUN domain in complex with the KASH domain peptide derived from nesprin-1 and nesprin-2 [40,41]. Both research groups used the SUN2 protomer SUN2_522–717_, consisting of a SUN domain and a minimal helical coiled-coil region (SUN2_525–540_), which are necessary for SUN2 trimerization and KASH binding [40]. Of note, this helical domain (SUN2_525–540_) is outside of the previously defined coiled-coil region (see below). The overall conformation of the SUN domain in complex with the KASH peptide closely resembles the SUN domain homotrimer in its apo state: a trimeric SUN2 structure with a globular head composed of the SUN domain, and a stalk composed of a triple helical coiled-coil (Figure 1A). Three SUN domains interact with three independent “hook”-like KASH peptides, forming a 3:3 hexameric heterocomplex (Figure 1B). The SUN domain consists of a beta-sandwich core and a ~20-amino acid beta-hairpin (SUN2_567–587_) extending as a long flexible loop, termed the KASH lid [39]. Each KASH peptide interacts with the KASH lid of one SUN2 protomer and the beta-sandwich core of the neighbouring SUN2 protomers. Thus, three KASH peptides effectively interconnect with multiple SUN proteins making up the 3:3 hetero-hexamer [40,41]. These results indicate that multimerization of SUN monomers through the triple helical coiled-coil is required for the KASH binding. In addition, disulphide bonds between conserved cysteines on SUN and KASH covalently link the SUN–KASH complex [40,43]. Though these disulphide bonds have not been detected in cells, molecular simulation suggested that disulphide bonds are crucial for the stability of the complex and the transmission of forces through the complex [42].

It is assumed that the characteristic cloverleaf-like and trimeric SUN arrangement is universally conserved for all mammalian SUN proteins through SUN1 to SUN5 for the following two reasons. First, the amino acid sequence of the SUN domain is well-conserved across divergent species, and all SUN domains are immediately preceded by the predicted coiled-coil segments [40]. Second, the trimeric arrangement of the SUN domain is a prerequisite for the KASH binding. The homotrimer formation for all mammalian SUN proteins is highly possible, but the regulation and stability of SUN3–5 may be substantially different from those of SUN1 and SUN2. This is because the latter proteins in mice and humans contain the cysteine residue that can form disulphide bonds with four nesprin family proteins, and their coiled-coil domain is predicted to be ~40 nm in length, whereas the germ cell-specific SUN3, SUN4, and SUN5 proteins have a shorter coiled-coil domain and do not contain the conserved cysteine residue [39,40,42]. By contrast, in nematodes, yeast, and plants, the sequences are quite divergent, and in particular, in Sad1 from *S. pombe*, the KASH lid is hardly recognizable according to its sequence and the typical disulphide bonds are lost [40]. Therefore, experimental data are needed to determine the conserved SUN protein trimer formation for other species.

## 3. The Coiled-Coil Domain in SUN Proteins Helps to Control the KASH Binding Capacity

All known SUN protein homologs from divergent species contain at least one coiled-coil segment in the luminal region near the amino-terminal end of the SUN domain. Coiled-coil domains have been generally regarded as oligomerization centres for the assembly of supramolecular protein complexes [44]. Coiled-coil domains are also found in several structural proteins that show highly elastic properties, suggesting that the SUN1 and SUN2 proteins are suitable elastic load-bearing components under the constant application and release of cytoskeletal forces to the NE [45].

In addition, coiled-coil domains are also believed to act as rigid spacers to define the distance between the ONM and INM of the NE [11,46]. The full coiled-coil region of the SUN1 and SUN2 proteins is predicted to be ~40 nm in length, which is similar to the distance between the ONM and INM [40]. Electron microscopy analysis using HeLa cells showed uniform spacing between the ONM and INM of ~50 nm; however, in the double SUN1 and SUN2-depleted cells, the ONM was clearly dilated with obvious expansion of the PNS to 100 nm or more [11]. It is worth noting that another mechanism likely exists to maintain the space between the ONM and INM in *C. elegans* [47].

In addition to the above predicted functions, the coiled-coil domains of SUN proteins have been shown to play roles in the regulation of SUN domain activity. A recent crystal structure analysis demonstrated that the two coiled-coil domains of SUN2, named CC1 and CC2, exhibit two distinct oligomeric states [43]. CC1 and CC2 are the distal and proximal coiled-coil domains with respect to the SUN domain, respectively. CC2 forms a three-helix bundle to lock the SUN domain in an inactive conformation, and sequesters the KASH lid of the SUN domain that is essential for anchoring the KASH domain in the SUN–KASH complex. In contrast, CC1 is a trimeric coiled-coil for the trimerization and activation of the SUN domain. Therefore, the two coiled-coil domains of SUN2 act as intrinsic dynamic regulators [43]. The results of solution binding assays are consistent with these structural analysis results for CC1 and CC2; removal of the CC1 of SUN1 or SUN2 abrogated their interaction with nesprins, whereas these deletion mutants retained the minimum region required for KASH binding [11,39,41,48,49]; i.e., the SUN domain and upstream extension consisting of the CC2 domain without CC1 suppressed trimer formation. Collectively, these findings demonstrate that the coiled-coil motif in the SUN protein does not simply function as a passive linear coiled-coil for oligomerization but further regulates SUN–KASH (de)coupling through the modulation of SUN domain oligomerization. However, it remains unknown how the wild type SUN protein, which possesses both CC1 and CC2, regulates the trimerization under physiological conditions. A clue to resolving this question was provided by Nie et al. [43], who reported that a SUN protein fragment containing CC1, CC2, and the SUN domain exists in a monomer- and trimer equilibrium state.

## 4. Compositional Nature of the SUN–KASH Hetero-Hexamer

**Figure 2:**
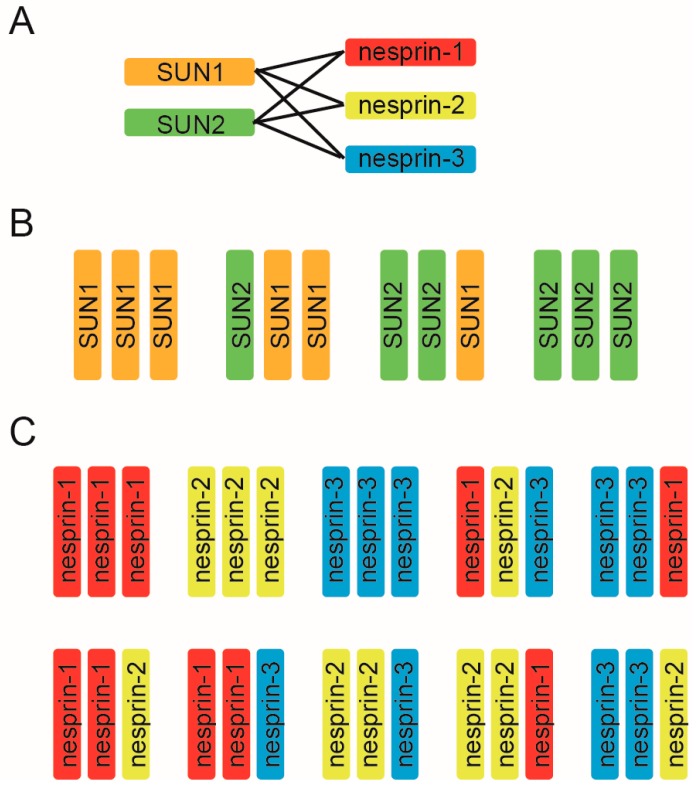
The compositional nature of the SUN–KASH hetero-hexamer. (**A**) The SUN–KASH combination is promiscuous. In mammalian somatic cells, except for the secretory epithelia, there are six combinations possible for two SUN proteins and three nesprins; (**B**) Mammalian cells mainly express SUN1 and SUN2 protein in somatic cells. SUN1 and SUN2 homo- and hetero-trimeric arrangement is conceivable; thus, four combinations of SUN trimers are possible; (**C**) Possible combinations of nesprin protein trimers using nesprin-1, nesprin-2, and nesprin-3, which are expressed in mammalian somatic cells.

Mammalian somatic cells, excluding epithelial cells, express two kinds of SUN, (SUN1 and SUN2) and three kinds of nesprin proteins (nesprin-1, nesprin-2, and nesprin-3) and it has been believed that each LINC complex is composed of three SUN and three nesprin molecules based on the crystal structure. Thus, to uncover the molecular mechanism underlying the diverse LINC complex functions, it is essential to first understand the compositional nature of the SUN–KASH hexamer from the aspect of two key points: the SUN–KASH interaction and the compositions of trimers. First, LINC complex formation relies on the direct binding of two kinds of SUN molecules and three kinds of KASH molecules. Long-standing solution binding assays have shown that both SUN1 and SUN2 interact with all of nesprin-1, -2, and -3, and vice versa, suggesting promiscuous interactions between the SUN and nesprin protein families [11,48,49,50,51]. Functional analyses support this promiscuous interaction; SUN1 and SUN2 are redundant in their anchoring functions of nesprins at the NE, and deletion of either SUN1 or SUN2 alone does not disrupt LINC connections [11,48,49]. In addition, the results of structural analyses further support such promiscuous interactions. The crystallographic structures of SUN2–KASH1 and SUN2–KASH2 look very similar; a hydrophobic pocket on the surface of the SUN domain serves as the docking site for the KASH domain PPPT motif, which is highly conserved in all nesprin proteins [40,42]. Therefore, the SUN–KASH combination is indeed promiscuous, and six combinations exist for interactions of the two SUN proteins and three nesprins (Figure 2A). It is to be noted that two SUN and four nesprin proteins are promiscuously able to bind each other, however it is not always true that deletion of either SUN alone has no effect on LINC complex functions. For example, SUN2 deletion alone affects nuclear movement in polarizing fibroblasts [35]; SUN1 knockout showed neuronal defects in the brain [30,52]; SUN1 is required for germ cell development [53]; and SUN2 knockout causes defects in skin and hair [54]. These facts again stress the SUN-KASH combination is critical for the LINC complex functions.

To add further complexity to the matter, in addition to the promiscuous SUN–KASH combinations, SUN and KASH can form a 3:3 hetero-hexamer. The LINC complex may include several combinations of SUN trimers (Figure 2B) and nesprin trimers (Figure 2C). As well as the above-mentioned SUN2 homotrimer demonstrated by crystallographic analysis, a SUN1 homotrimer should also exist [40,55]. Moreover, SUN1–SUN2 heterotrimer formation is feasible for the following two reasons. First, biochemical analyses have shown that SUN1 and SUN2 can form a hetero-oligomer [11,28,49,55,56,57], and second, the molecular organization of SUN 1 and SUN2, such as the length of the coiled-coil domains or the SUN domain, is similar. Thus, four kinds of SUN protein trimers are possible (Figure 2B). By the same logic, 10 nesprin trimers are possible from the combination of three nesprins (Figure 2C). Collectively, these combinations would result in a whopping total of ~40 combinations of SUN and nesprin hetero-hexamers. However, this prediction represents a great simplification of the actual situation. We have no evidence for that there are always three KASH domain proteins bound to a SUN trimer. It might be possible that SUN/KASH domain proteins can not only interact at a 3:3 ratio, but also at 3:1 and 3:2 ratios. Likewise, SUN1 may exist in dimers or tetramers, not trimers in cells under some circumstances [55]. In addition, since each gene encoding SUN and nesprin proteins produces a wide range of splicing variants, and the expression level of variant proteins is tissue-dependent, the compositional nature of SUN–KASH hexamers significantly varies among tissues [10,11,12,21,28,58].

**Figure 3:**
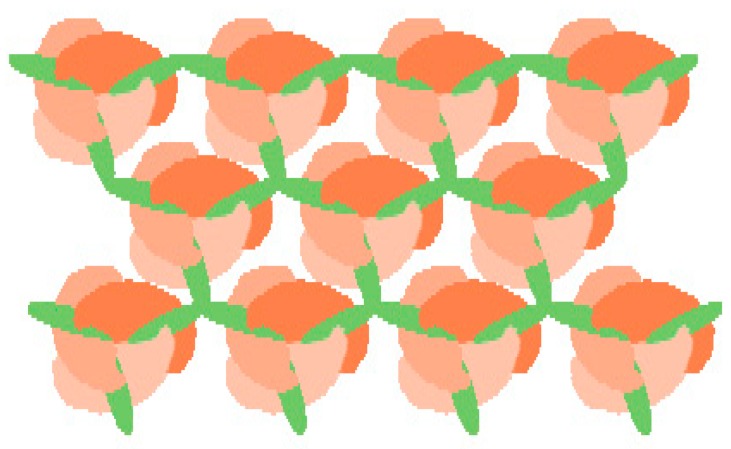
Multivalent SUN–KASH interactions. The interaction of nesprin with neighbouring SUN trimers enables the formation of higher-order complexes; i.e., the association of the KASH domain from trimeric/oligomeric nesprins with neighbouring SUN trimers allows for higher-order arrays of the LINC complex. Orange and green molecules represent SUN and KASH peptides, respectively.

There is one other key factor to keep in mind. Several reports have demonstrated that some KASH proteins are organized by multimerization [50,59,60]. However, at present, the organization of KASH proteins is unclear, and it is also unknown whether the three SUN domains in a trimer interact with the KASH domains originating from individual KASH protein monomers, from a single nesprin trimer, or from even higher organizational units (Figure 1A). Interestingly, it has been suggested that the transmembrane helices of nesprins might engage in protein–protein interactions [40]. Based on crystallographic structural data, Sosa et al. [40] indicated that the transmembrane helices of KASH proteins cannot interact with each other in the membrane if all of the peptides comprising a KASH oligomer are bound to SUN domains from a single SUN trimer. This is because the three KASH peptides are ~50 Å apart on a SUN trimer (see Figure 1B) and the transmembrane helix is quite close to the KASH domain (with only seven amino acid residues separating them). They proposed an attractive model in which self-association of the transmembrane helix in nesprins would prevent the interaction of nesprins with the same SUN trimer, which would instead interact with neighbouring SUN trimers, thus enabling the formation of higher-order complexes (Figure 3).

The molecular mechanism underlying the higher-order assembly of the LINC complex has not been elucidated; however, the existence of such structures is evident in at least two physiological situations. First, SUN proteins can form higher-order oligomers or clusters during meiosis in various organisms. Fission yeast SUN protein, Sad1 and its KASH partner, Kms1, function in chromosomal bouquet formation during meiosis which facilitates homologous chromosome pairing. In *C. elegans*, SUN protein and the KASH partner, ZYG-12, also make large complexes in meiosis. Similar aggregates are found in mouse meiosis [50,59,60,61,62,63,64,65,66]. Second, transmembrane actin-associated nuclear (TAN) lines, which are linear actin-associated arrays of SUN2 and nesprin-2G, are formed to reorient the nucleus during cell migration [35,67]. To clarify the LINC complex composition, the organization of the KASH protein oligomer must first be solved at the crystallographic structure level.

## 5. Lamins Interact with SUN Proteins and Affect Their Dynamics

The *LMNA* gene encodes the two major A-type lamins, lamin A and lamin C, whereas *LMNB1* and *LMNB2* encode the two major B-type lamins, lamin B1 and lamin B2, respectively [68,69,70,71]. The nucleoplasmic region of both SUN1 and SUN2 interacts with lamin A, whereas the interaction with B-type lamins appears to be relatively weak [11,16]. However, as mentioned above, a recent solution binding assay and yeast two-hybrid analysis revealed that both lamin B1 and lamin B2 interact with SUN1 but not with SUN2 [28].

A-type lamins could contribute to SUN2 localization, although they are certainly not the only determinants [11]. Studies with *LMNA*-null (*LMNA*^−/−^) mouse embryonic fibroblasts (MEFs) showed that SUN2 was dispersed throughout the cytoplasmic membranes in the majority of cells, with a minority showing SUN2 fully retained at the NE. In contrast, lamin A/C is not required for localization of SUN1 in the INM [11,16,72]. However, the interactions with lamin A/C affect both SUN1 and SUN2 protein dynamics [51]. SUN1 and SUN2 are more mobile in *LMNA*^−/−^ MEFs than in wild-type MEFs. In addition, fluorescence resonance energy transfer (FRET) experiments showed that SUN1 is more closely associated with lamin A than SUN2, suggesting a higher affinity for SUN1 [51]. These findings may provide an explanation for the observation that overexpression of SUN1 in HeLa cells causes displacement of endogenous SUN2 from the NE, while the converse is not the case. In addition, from the point of SUN INM anchoring, it is worth mentioning that mouse SUN1 possesses a zinc finger motif and SUN protein in *Dictyostelium*, which does not have lamins, interacts with DNA [57].

SUN proteins and lamins clearly interact in coordinated ways; however, the mechanisms controlling these interactions remain largely unknown. One possible regulatory mechanism of this association is post-translated modification. Since both lamins and SUN proteins undergo various post-translational modifications such as phosphorylation and sumoylation [73,74,75], these modifications might affect and regulate LINC complex–lamins interactions. One well-investigated example is mitosis-dependent disassembly. Since the nucleus breaks down during mitosis in multicellular organisms, the complex of SUN and lamins is inevitably disassembled at least once in each cell cycle. During mitosis, SUN proteins play an active role in NE breakdown [76]; phosphorylation of lamin and SUN proteins induces the dissociation between these molecules while the SUN–KASH interaction is retained, indicating that phosphorylation regulates the lamin-LINC interaction but lamin A/C is not essential for SUN–KASH binding at least during mitosis [74].

## 6. Possible Functions of Lamins–SUNs Interactions

The human *SUN1* gene produces over 10 alternative splicing variants that are distinguished by variable deletions just upstream from the transmembrane domain, between exon 6 and 9 [28]. Thus, all of the splicing variants contain a lamin A-binding domain, which is located at the extreme amino-terminal domain of the SUN protein [11,12,28]. A solution binding assay confirmed that lamin A interacted with all of the investigated SUN1 splicing variants as well as with SUN2 [28], suggesting that the lamin A–SUN interaction may play fundamental roles such as in the regulation of KASH binding. In contrast, in the same experiment, B-type lamins interacted with the SUN1 variants but not with SUN2 [28]. Although the B-type lamins-binding sites are not yet known, this result suggests that the interaction with B-type lamins plays a role in SUN1-specific functions such as cerebellar development, nuclear pore complex organization, or chromatin tethering.

Another key question to help understand the function of the LINC complex is whether or not the interaction between SUNs and lamins affects the LINC complex formation such as structure, composition, and combination. It is well known that SUN1 and SUN2 have a redundant function in nesprins ONM localization. In addition, nesprin-2 was relocalized to the endoplasmic reticulum in *LMNA*^−/−^ cells [58], indicating a role for lamins, in addition to SUNs, in the localization of nesprins to the NE in interphase cells. Because lamins are physically separate from nesprins, and disruption of the SUN trimer abolished KASH binding [39], the association of SUNs with lamins might affect the overall structure of SUN proteins in interphase cells, resulting in regulation of the SUN–KASH interaction. It is attractive to speculate that the interaction with lamin might be the molecular switch for SUN-KASH oligomer formation, but this idea awaits experimental verification.

## 7. Pathological Relevance of the Lamins–SUNs Interplay

Mutations in the *LMNA* gene encoding A-type lamins cause a wide range of diseases, including muscular dystrophies, lipodystrophy, and progeria, which are collectively referred to as laminopathies [38]. Intriguingly, some nesprin gene mutations also induce similar phenotypes. For example, Emery-Dreifuss muscular dystrophy, which affects the skeletal and cardiac muscle, is commonly caused by mutation in the emerin (*EMD*) gene and less frequently by mutation in *LMNA*, and has also been associated with a mutation in the nesprin gene [77,78]. Nevertheless, the pathological relevance of the LINC–lamins interplay remains largely unknown. One possible reason for these etiologies is that mutations in the genes encoding A-type lamins or *EMD* might specifically weaken, prevent, or strengthen the SUN–lamin–emerin interaction, which would also affect the physical connections between SUN and KASH proteins, resulting in altered associations between the nuclear lamina and cytoskeleton [79,80]. Thus, the disruption of adequate interactions between SUNs and lamins could be ultimately responsible for the pathological effects.

In Hutchinson-Gilford progeria syndrome (HGPS), the most common mutation is a de novo missense mutation in exon 11 of the *LMNA* gene, which results in the creation of an abnormal splice donor site and expression of a 50-amino acid region near the carboxy terminus to result in a truncated protein, permanently farnesylated prelamin A, termed progerin [81,82]. Progerin has higher affinity to SUN1 than lamin A [83]. Thus, accumulated progerin interacts with SUN1 and induces the accumulation of SUN1, which contributes to the nuclear aberrancies in HGPS. As another example, mandibuloacral dysplasia type A is caused by a recessive mutation of the *LMNA* gene and is a rare laminopathy characterized by several skeletal and tissue defects, including postnatal growth retardation, craniofacial anomalies, and bone resorption at specific sites [84]. This mutation induces the accumulation of the unprocessed prelamin A precursor and also alters SUN1 and SUN2 localization.

Besides these disease-linked effects, mutations of the *LMNA* gene induce global epigenetic defects. Human fibroblast cells derived from both HGPS and mandibuloacral dysplasia type A patients showed altered histone H3 lysine 9 (H3K9) methylation status [85], and experimentally altered H3K9 methylation status induced SUN2 protein disorganization (Hieda et al., unpublished data). These observations could be directly induced by aberrant lamin A protein expression but could also be induced by unusual epigenetic status.

## 8. Perspectives

The functions of the mammalian LINC complex have been studied for nearly a decade now, and progress in this field has accelerated in recent years. In particular, the atomic resolutions of the SUN domain provide valuable insights into the functional diversities of LINC complexes and give plausible interpretations for a decade-old biochemical observation. However, this research has also raised many new questions. For example, do the LINC complex linkages disassemble despite the tight association of SUN and KASH, and if so, how? How often does the LINC complex interchange? The trimeric formation of the SUN domain is the prerequisite for the mode of KASH binding; thus, the regulation of trimerization may control SUN–KASH complex formation. If so, how is the equilibration between the SUN trimer and monomer regulated? What are the functions of lamin–LINC complex connections? How is the timing of the lamin–LINC complex interaction regulated? How much and how often do SUNs and lamins interact? Finally, could the interaction between SUNs and lamins affect compositional nature of SUN/KASH oligomer?

Disconnection of LINC complexes disrupts the flow of physical and molecular information between the two cell compartments inside and outside of the nucleus. Therefore, many recent studies of LINC complexes have focused on the information transfer from the cytoplasm into the nucleus. However, I would like to emphasize the possibility that lamins–SUNs connections might play roles in controlling LINC complex functions, and it is highly possible that the LINC complex can bidirectionally transfer information between the cytoplasm and the nucleus, similar to the role of plasma membrane receptors such as integrins. Finally, resolving the above fundamental questions related to LINC complex–lamins interactions will potentially help to explain the overall functions of the LINC complex, and highlight new therapeutic targets for treating laminopathies.

## Abbreviations

HGPS	Hutchinson-Gilford progeria syndrome
INM	inner nuclear membrane
LINC	linker of nucleoskeleton and cytoskeleton
MEF	mouse embryonic fibroblast
NE	nuclear envelope
ONM	outer nuclear membrane
PNS	perinuclear space
